# Detecting changepoints in dynamical systems: Modeling time-varying transmission of seasonal influenza

**DOI:** 10.1073/pnas.2533861123

**Published:** 2026-05-19

**Authors:** Ajay N. Oza, Katie M. O’Brien, James P. Gleeson

**Affiliations:** ^a^Respiratory Virus Unit, Health Service Executive-Health Protection Surveillance Centre, Dublin D01 A4A3, Ireland; ^b^Modelling and Biostatistics Unit, Health Service Executive-Health Protection Surveillance Center, Dublin D01 A4A3, Ireland; ^c^ Department of Mathematics and Statistics, Mathematics Applications Consortium for Science and Industry, University of Limerick, Limerick V94 T9PX, Ireland

**Keywords:** changepoint detection, forecasting, iterated filtering, influenza transmission

## Abstract

Seasonal influenza epidemics exhibit complex transmission dynamics influenced by time-varying extrinsic factors such as social behavior and seasonal effects. Estimating changes in transmission rates is critical to enable accurate forecasting of the epidemic curve. This study presents a framework for detecting changepoints in the transmission parameter ($\beta_{t}$), applied as a piecewise constant function within a deterministic compartmental model. Using hospitalized case data from four recent influenza A seasons in Ireland (2019/2020 and 2022–2025), we applied iterated filtering and kernel density estimation to identify season-specific and cross-seasonal changepoint structures. The algorithm integrates stochastic search, local perturbation, and resampling to infer the most plausible changepoint configurations. Results reveal consistent changepoint patterns across seasons, particularly during periods of increased social mixing, such as the December holiday period. A universal changepoint model was also developed, enabling medium-term forecasting and scenario planning. This approach offers a robust method for capturing abrupt shifts in transmission and may be applicable to other dynamical systems.

## Significance

Understanding how influenza transmission changes over time is critical for accurate forecasting and effective public health planning. Seasonal influenza does not spread at a constant rate, social behaviors, holidays, and environmental factors can cause shifts in transmission. This study introduces a modeling framework that detects these changes using advanced statistical techniques, including iterated filtering and kernel density estimation, applied to hospitalization data from multiple influenza A seasons. By identifying consistent changepoint patterns and season specific transmission rates, this approach improves our ability to anticipate epidemic peaks and design timely interventions, offering a robust tool for managing dynamic infectious disease systems.

Influenza A is a major respiratory infection of humans with annual epidemics occurring in temperate regions of the world (1). In Ireland and most of Europe the peak of the epidemic usually falls mid-winter, which, depending on the severity of the circulating viral subtype, can pose significant strain on hospitals and healthcare facilities (2, 3). Anticipating the likely trajectory of an epidemic can guide public health interventions and service planning.

Dynamical systems offer a structured framework to model infectious disease spread. Modeling approaches frequently use mechanistic methods such as compartmental SIR models that can be extended to include hospitalized cases (4, 5). However, time-varying extrinsic factors that are critical to the epidemic course, such as social behavior cycles and seasonal effects, pose a calibration problem for estimating the model parameters, in particular the transmission parameter, usually denoted as $\beta_{t}$. Estimation methods such as those relying on effective reproduction number or diffusion process have been successfully applied (6, 7) however, interpretability of these, their robustness to noise and their flexibility in capturing abrupt changes remain a challenge (8).

Recently, generalized additive models (GAMs) have been used in conjunction with compartmental models to infer transmission parameters and reproduction numbers as smooth functions of time, described by splines. The criteria for inversion of compartmental models to determine the implied time-varying transmission are nontrivial (9) but such approaches can be successfully applied to various data, e.g., the impacts of SARS-CoV-2 in care home surveillance data (10). However, the use of smoothly varying spline functions makes it difficult to identify changepoints in time and regime shifts corresponding to nonpharmaceutical interventions.

Examples where the $\beta_{t}$ is modeled as piecewise linear function have emerged following investigations of the impact of effectiveness of nonpharmaceutical intervention for SARS-CoV-2 pandemic (11). A stepwise structure affords clearer interpretation of transmission regimes which could also be used for forecasting plausible scenarios.

**Fig. 1.**
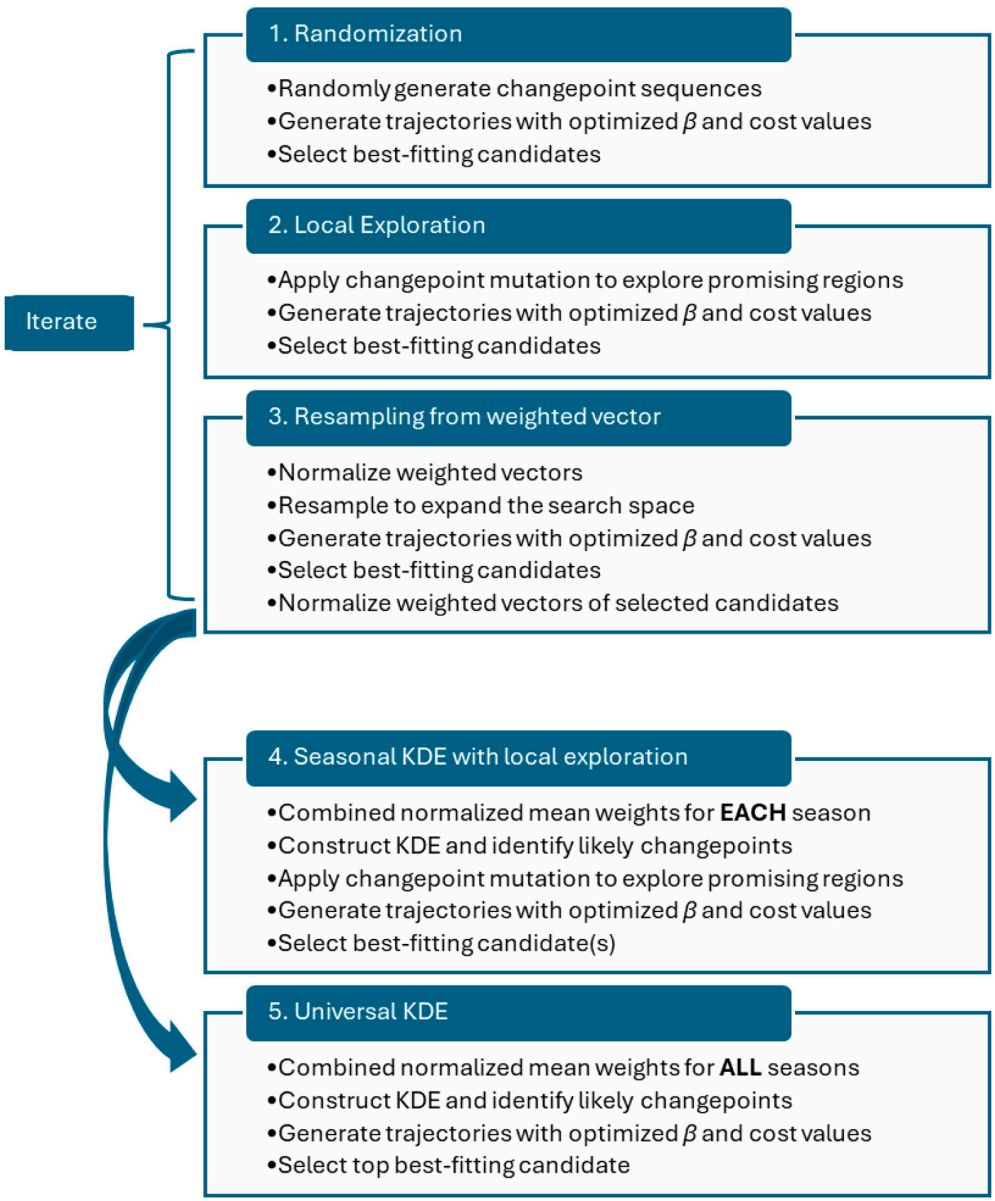
Flowchart of the main algorithmic approach to determine changepoints that demarcate the time-varying stepwise constant transmission parameter $\beta_{t}$. Data from four influenza A seasonal epidemics in Ireland were used.

In this work, we present a framework for detecting changepoints of $\beta_{t}$, which is modeled as piecewise constant over time. The method is designed to capture structural shifts in transmission dynamics, such as those driven by factors already mentioned. The algorithm incorporates stochastic search, local refinement, and kernel density estimation (KDE) to infer changepoint structures that are both season-specific and consistent across the multiple seasons of influenza A epidemics. We examine this changepoint detection methodology using data from hospitalized cases during four recent influenza A seasons from Ireland (Fig. 1).

## Results

**Fig. 2.**
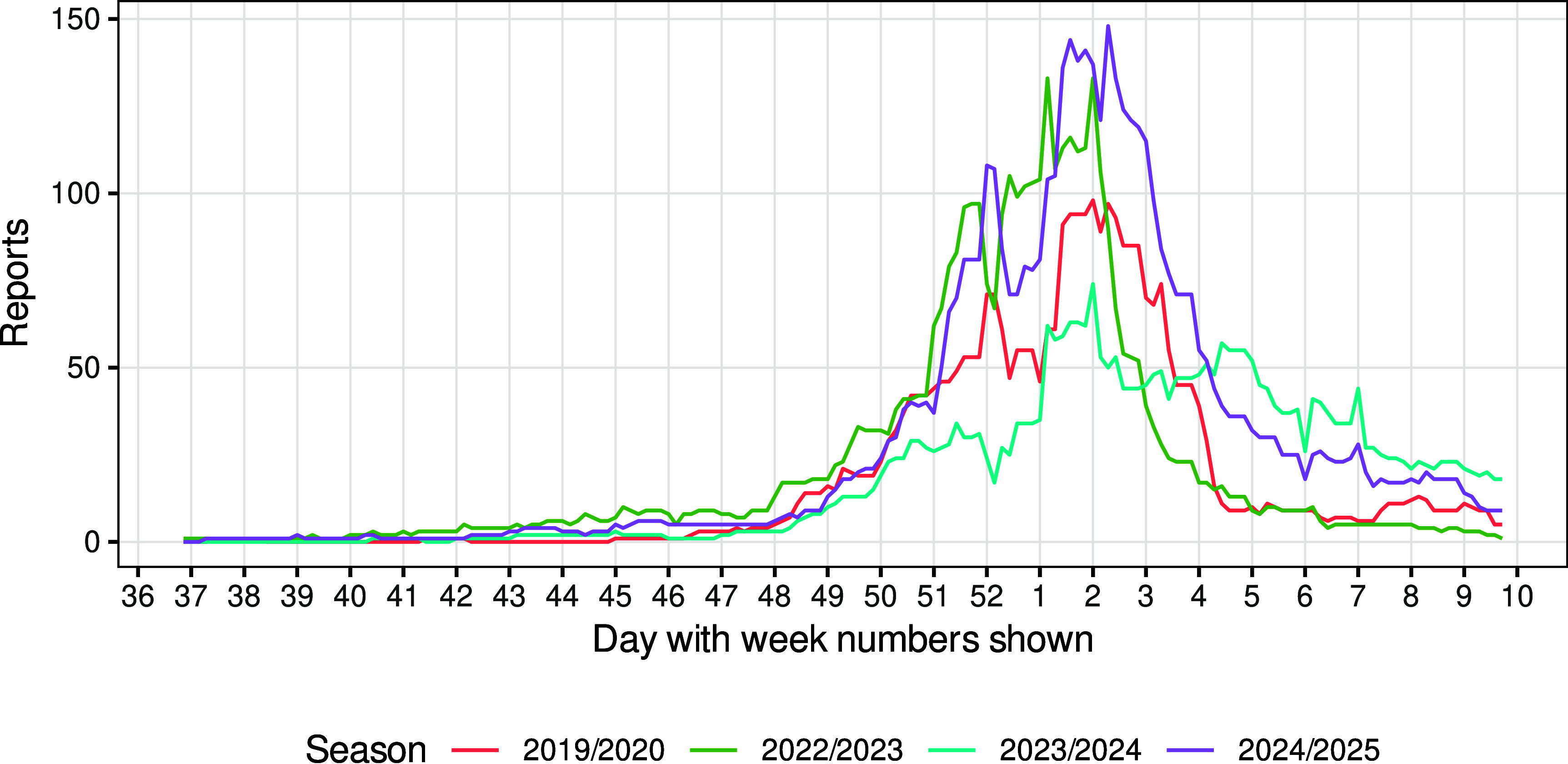
Time series of reports (7 d-moving average of influenza-A hospitalized cases) by day for each season studied.

The time series of the four seasons studied show a similar epidemic pattern (Fig. 2). In each season, the reports started to rise noticeably at the start of week 48 and peaked around week 1 or 2 of the following year. Week 52 exhibited underreporting, which is not uncommon in the northern hemisphere influenza season (12, 13). The amplitude differed between seasons, from around 75 reports in 2023/2024 to around 140 in 2024/2025.

**Fig. 3.**
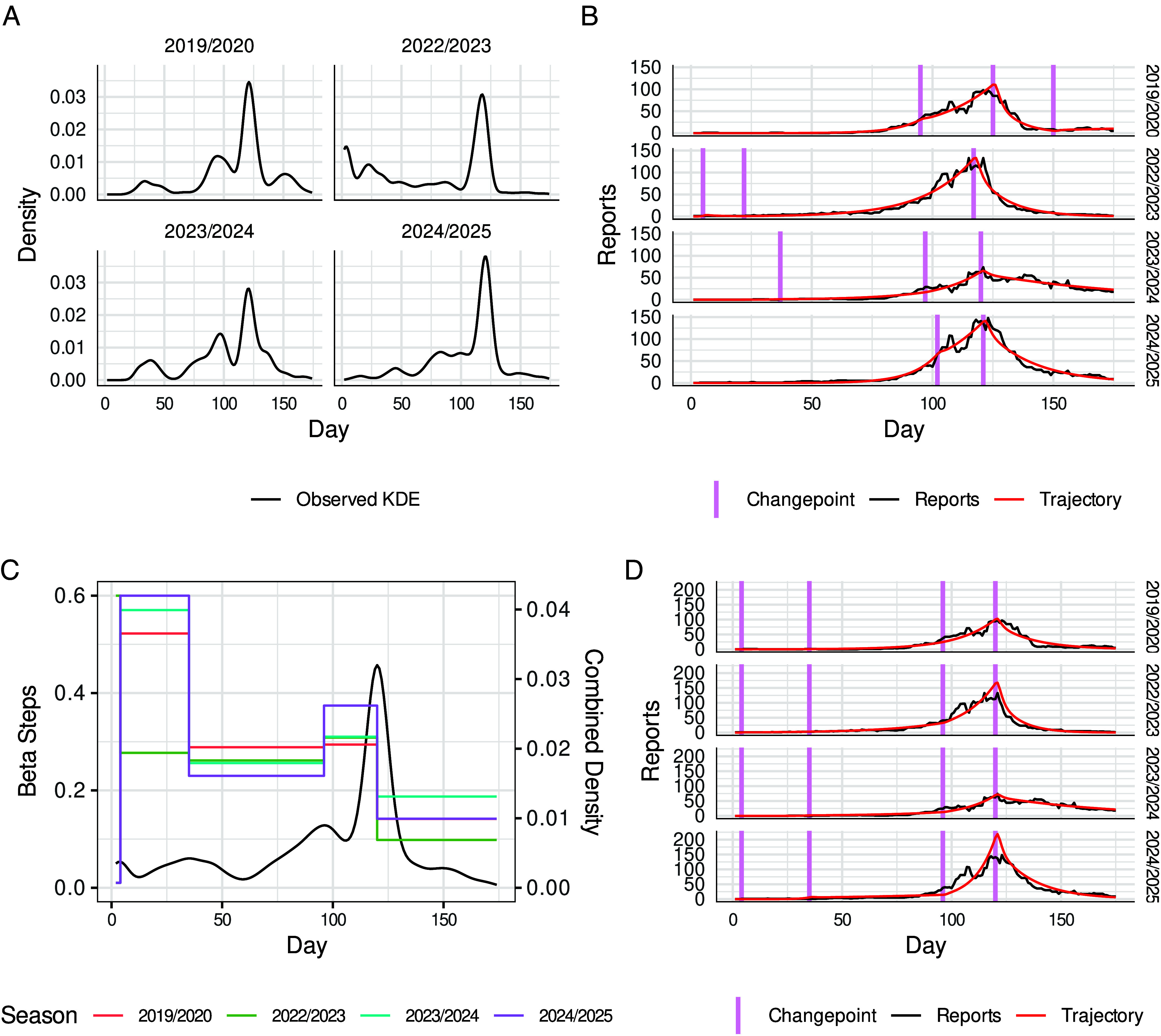
Season specific and universal changepoint locations shown, along with best fitting trajectories across four seasons based on optimized beta values. (*A*) Plots of kernel density estimations of the mean weights from the iterated filtering algorithm for finding season specific changepoints for piecewise constant transmission rate $\beta_{t}$. (*B*) Best fitting trajectories from the changepoint determination algorithm showing relative positions of changepoints across seasons. (*C*) Step functions for $\beta_{t}$ of the fitted trajectories for each season, along with the universal KDE. (*D*) Best fitting trajectories for each season based on optimized $\beta_{t}$ values with fixed changepoints as determined from the universal KDE.

The KDE smoothed distributions of changepoint likelihood across the four seasonal time series, based on the mean weights from 50 iterations, show peaks when changepoints were likely to occur (Fig. 3*A*). Across the different seasons, where day 1 occurs on Sunday of week 37 (approximately mid-September), a similar pattern of peaks (and hence plausible changepoints) was observed. In particular, there are peaks at around day 120 which corresponds to the second week of January already noted as the likely amplitude for each season. Additionally, peaks are commonly observed around day 100 or slightly earlier. The 2022/2023 season uniquely showed peaks in the initial part of the season.

These patterns provide an insight into both the commonality across seasons and the season-specific nature of the changepoints, which is further demonstrated through the best fitted trajectories (Fig. 3*B*).

Fig. 3 *C* and *D* present the results of the universal KDE analysis, combining data across all seasons to identify common changepoint structures. Rather than identifying changepoints independently for each season, this final stage imposes a common set of changepoints onto each season time series. The resulting $\beta_{t}$, expressed as step functions over the changepoint intervals, reveal a notable degree of similarity in the magnitude and direction of change across seasons. However, the first interval exhibited pronounced differences, suggesting that while the universal changepoints capture broadly shared temporal features, the underlying seasonal responses remain distinct in certain cases.

The $\beta_{t}$ values for the intervals between changepoints are summarized in Table 1. The interval starting at day 1 had the highest variability largely driven by the high value for 2022/2023 season. The $\beta_{t}$ values are more consistent for the interval starting at day 4 and relatively high for the other seasons. Together the $\beta_{t}$ values for the first two intervals may reflect the differences in epidemic for a transient period and onset between seasons. The decrease in mean values between interval two (0.507, 4 to 34 d) and three (0.259, 35 to 95 d, starting week 42) may reflect a period of routine, settled social mixing while the number of cases gradually increase over the course of about 8 wk. The increase in infections for interval 4 (mean 0.322, 96 to 119 d, starting week 50), which was consistent across the seasons, corresponds to a period of increased social mixing and the start of the holiday period in December, and the epidemics are noted for rapid growth at this time. The final interval (0.142, 120 to 175 d, week 2) corresponds to the end of the holiday period.

**Table 1.** Intervals between universal changepoints and their associated optimized $\beta_{t}$ values for each season

	Intervals between universal changepoints				
	1–3	4–34	35–95	96–119	120–175
2019/2020	0.010	0.522	0.289	0.294	0.142
2022/2023	4.000	0.277	0.262	0.308	0.098
2023/2024	0.010	0.570	0.256	0.310	0.187
2024/2025	0.010	0.658	0.230	0.374	0.142
**Mean** $\beta_{t}$	–	**0.507**	**0.259**	**0.322**	**0.142**
**Start Dates**	8th Sept	11th Sept	12th Oct	12th Dec	5th Jan
**Label**	Transient	Onset	Build-up	Take-off	Release

The average of the $\beta_{t}$ values across the four seasons is also shown along with start dates for each interval for 2024/2025.

### Cost Analysis across Seasons and Stages of the Main Algorithm.

Each candidate set of potential changepoints at each sampling and iteration were used to optimize $\beta_{t}$ values, and the generated trajectories’ cost (negative likelihood) were used for selection and further analysis. Refer to Fig. 1 for a brief description of the stages.

**Fig. 4.**
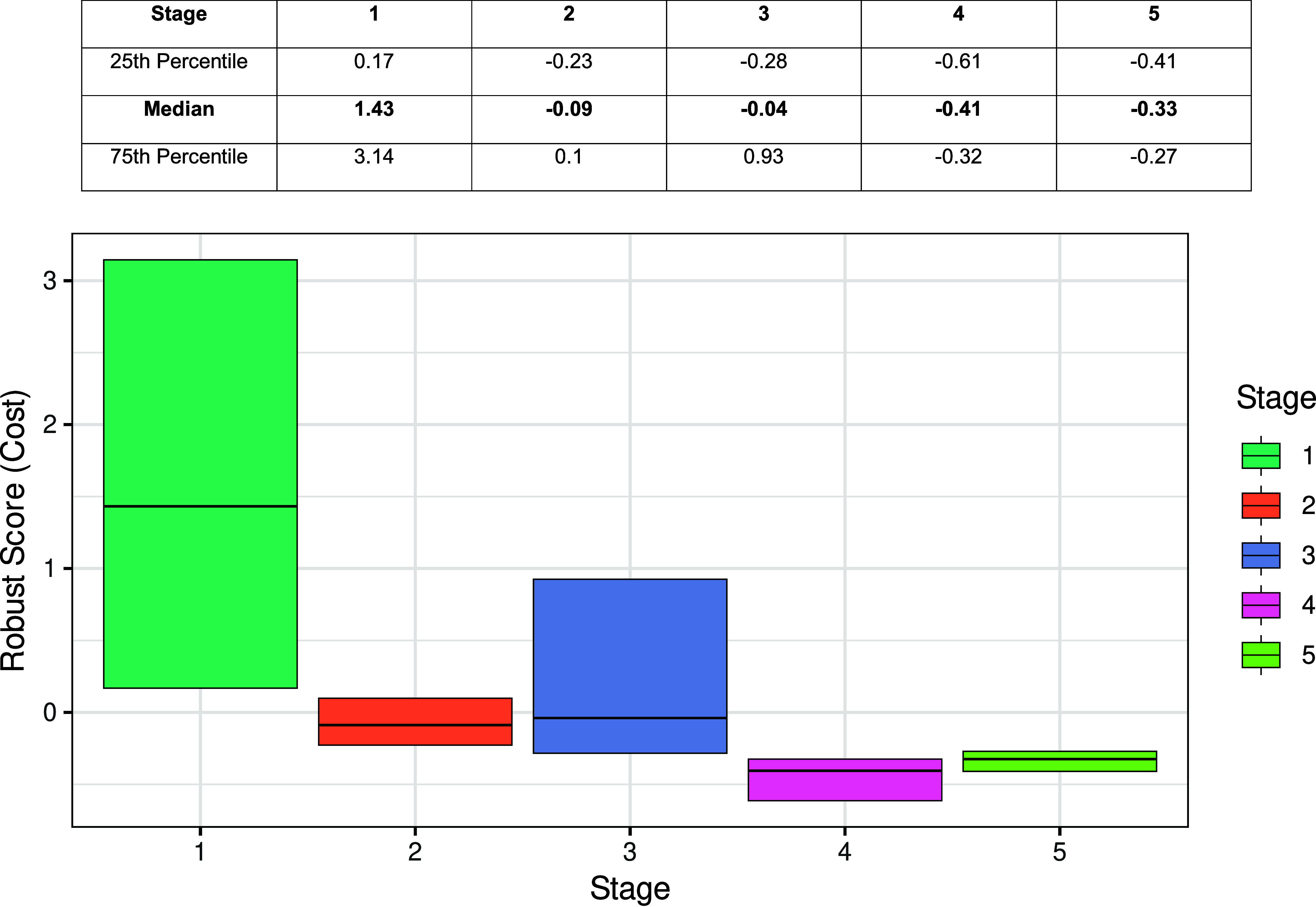
A simplified box plot of adjusted robust cost scores associated with the fitted trajectories for each stage, summary of adjusted robust cost scores for each stage in adjacent pane.

The interquartile ranges for each stage remain broadly consistent across seasons, but the contrast *between* stages is striking. For example, the median costs in stage 1 range from 2,619 to 2,081, whereas in stage 4 they fall to just 422 to 677. This is clearly owing to the difference in the underlying observed data. To better compare the changes between stages, an adjusted robust score was used in the following way. For each season, the worst-performing (i.e., highest cost) 10% of the candidates, regardless of stage, were excluded. The median was subtracted from each cost value, and the result was divided by the interquartile range (IQR) for that season (14). The outputs are shown in Fig. 4. This analysis enables a discussion of the stage-by-stage distribution of costs, as follows.

Stage 1: Randomization. This stage yielded the highest adjusted cost values, both in terms of median (1.43) and IQR (0.17 to 3.14). This is expected, as the changepoint sequences to initialize Stage 1 are randomly generated without prior knowledge or refinement. The wide IQR reflects the diversity of candidate solutions, the majority of which are suboptimal.

Stage 2: Local Exploration. Here, the median adjusted cost (−0.09) is lower than the value from Stage 1, and the IQR narrowed sharply (−0.22 to 0.10). This suggests that local mutation around promising candidates effectively refined the changepoint configurations, concentrating the search in regions of lower cost and higher fit.

Stage 3: Resampling from Weighted Vector. The median adjusted cost increased slightly (−0.04) relative to Stage 2, and the IQR widened again (−0.28 to 0.92). This stage reintroduces diversity by resampling from a weighted pool, expanding the search space. While this can uncover new promising regions, it also brings in more variability, hence the broader IQR.

Stage 4: Seasonal KDE with Local Exploration. This stage achieved the lowest median adjusted cost of all analytical stages (−0.41), indicating that leveraging season-specific changepoint likelihoods and refining them locally is highly effective. The KDE helps concentrate the search around structurally consistent changepoints, improving fit across seasons.

Stage 5: Universal KDE. The median adjusted cost (−0.33) increased slightly compared to Stage 4, though it remained lower than earlier stages. This stage generalizes across all seasons, which may dilute season-specific signals. The slight increase could reflect the challenge of balancing fit across diverse seasonal dynamics.

## Discussion

To investigate temporal structure across multiple seasons of influenza A epidemics, we conducted a cross-seasonal analysis of changepoint likelihoods. While our approach does not aim to pinpoint exact transmission rate changepoint locations, it focuses on estimating the likelihood of changepoint occurrence at each point within seasonal epidemic trajectories. By aggregating these likelihood profiles across multiple seasons, we were able to identify periods with consistently elevated changepoint probabilities, offering insight into shared structural dynamics and potential drivers of change across seasons. Interpretation of changepoints and synthetic data experiments to explore the methodology is further discussed in *SI Appendix*, Text section B.

The iterated filtering approach was designed to efficiently navigate the vast combinatorial space of possible changepoint configurations, where brute-force enumeration would be computationally infeasible. By seeding the process with randomly generated candidate sequences and iteratively refining them, the method avoids the pitfalls of local optima that can arise in some greedy search strategies (15). Reinitialization through multiple iterations ensures that the search does not become trapped in suboptimal regions of the solution spaces. The perturbation step plays a crucial role by introducing controlled alterations around promising changepoint locations, allowing the algorithm to explore local neighborhoods more thoroughly. Furthermore, the resampling of candidates based on weighted likelihoods broadens the search space, enabling the identification of high-performing configurations that may not have been captured through local exploration alone. This layered strategy, akin to a genetic algorithm (16), balances exploitation of promising regions with exploration of the wider space, ultimately yielding robust estimates of changepoint likelihood across the time series.

The rationale for the final two stages was to assess the consistency of inferred changepoint structures across seasons and to explore the potential for a shared temporal framework. KDEs of changepoint weights were used to identify the most likely changepoint locations within each season. This approach allowed for a nuanced comparison of changepoint patterns across seasons, highlighting both convergence and divergence in temporal structure. The universal KDE was constructed by changepoint weights across all seasons, enabling the identification of changepoints that were most consistently supported across the four time series. These universal changepoints were then imposed on each season, and $\beta_{t}$ values were optimized individually to assess how well a shared changepoint structure could accommodate season-specific dynamics.

The analysis of the progression of cost values across the stages of the algorithm (Fig. 4) reflects a deliberate balance between exploration and exploitation. Early stages prioritize diversity, while later stages focus on refinement and structural consistency. The reduction in adjusted cost value at Stage 4 highlights the strength of incorporating season-specific information, whereas the slight rise in Stage 5 suggests that universal patterns may not always outperform season-specific refinements.

The analysis of $\beta_{t}$ values across universal changepoint intervals reveals meaningful temporal structure in transmission dynamics. Early season variability likely reflects differences in epidemic onset from year to year. The subsequent rise during the holiday period underscores the influence of social mixing on epidemic acceleration. Together, these patterns highlight the utility of the stepwise constant $\beta_{t}$ framework in capturing both shared and season-specific drivers of influenza (17), agreeing with the observation that influenza seasons exhibit both recurrent and unique patterns of spread. While some studies have considered season specific analysis, (18–20) the current study extends to a cross-seasonal approach.

We limited our investigation to a maximum of four changepoints. Given the 175-day observation window (i.e., 175 potential changepoints), this may be insufficient to fully capture the subtle dynamics of the epidemic curve. However, this constraint helps prevent overfitting, which is particularly important as our goal is to apply this method for national-level influenza forecasting.

Our analysis includes three post-COVID-19 seasons and one pre-COVID-19 season. We excluded earlier seasons due to changes in testing (21) such as the introduction of near-patient testing, which limit the comparability of older data for model training (22).

Currently, the model is calibrated to all-cause hospital admissions with a positive influenza test result, rather than community-level data. Given the underreporting of influenza cases, this approach was deemed more robust. However, future work could benefit from triangulating with additional data sources, such sentinel acute-care (23) and primary-care surveillance (24, 25). This could also be used to incorporate another time-varying parameter; the proportion of cases which are hospitalized. This can vary during the season, when hospitals reach or are close to capacity.

The methods described in this paper can be used to monitor and provide short/medium term forecasts of new hospitalized influenza A cases and thus feed into winter planning within the health system with respect to hospital utilization (26). The methods may also be applied to other pathogens where appropriate. Additionally, resampling from the KDE can yield synthetic datasets, which can be used as training data or for sharing data where disclosure of the raw data may be sensitive (27).

Although the approach described here has focused on application to a deterministic compartmental model, the changepoint methodology could potentially also be applied to other dynamical models for epidemics. Hawkes processes (28), for example, may have altered parameter values due to shifts in transmission, and the detection of these changepoints–especially in Hawkes models that account for the finite size of the susceptible population (29)–is a promising direction for extensions of this work.

## Conclusion

Prior to the onset of an influenza season, information about the location of universal changepoints can help identify likely periods of change in the transmission parameter, $\beta_{t}$. These changepoint estimates can be updated dynamically as the season progresses and additional data become available. This approach offers the potential of a medium-term forecasting framework for influenza, incorporating current data and learnings from previous seasons.

## Materials and Methods

### Data.

Deidentified laboratory confirmed influenza case-based data notified on Ireland’s Computerised Infectious Disease Reporting (CIDR) system as collated by Health Service Executive - Health Protection Surveillance Centre (HSE-HPSC), were extracted on 05/05/2025 (30). Only data from samples that were taken from hospital inpatients were considered for this study. Cases that were confirmed as influenza with no virus typing data (0.1%), those typed only as influenza B (13.0%) or where the patient was coinfected with both influenza A and B (0.2%) were excluded (13.3% of total). Data from influenza season 2019/2020 (pre-COVID-19 pandemic) and three seasons following the COVID-19 pandemic (2022/2023, 2023/2024, 2024/2025) were analyzed. The period under consideration started on Sunday of week 37 (approximately mid-September) in the epidemiological calendar and extended to the end of week 9 (approximately late February/early March) of the following calendar year (Saturday). Thus, each season had days numbered 1 to 175. Daily reports were created by seven-day moving averages of case numbers by day for each season; data one week prior to day 1 were supplied as a burn-in period.

Hospital In-patient Enquiry (HIPE) data following patients’ discharge were used to obtain average length of stay, in days, for diagnosis codes matching influenza (J09-J11) (31).

### Model.

**Fig. 5.**
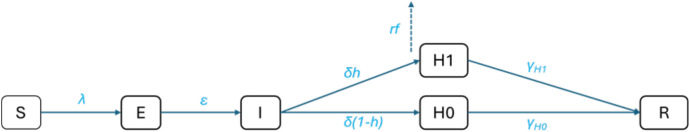
Flowchart of disease progression model.

We implemented a deterministic compartmental model using the R statistical package *pomp* (for partially observable Markov process) (32). The design was guided by studies that model hospitalized cases of respiratory viral diseases in particular (4, 33). The governing differential equations (Eqs. **2.1** – **2.4**), flow diagram (Fig. 5), and the states and the parameters (Table 2) are presented in this section.

**Table 2.** Parameters used in the model

Parameter	Description	Value
$\beta_{t}$	Time-varying disease transmission rate	Optimized
*λ*	Force of infection	Calculated
*ε*	Inverse of latent period (incubation rate)	0.5
*δ*	Progression rate	1
*h*	Proportion hospitalized	0.06
*γ*_*H0*_	Inverse of infectious period (average rate of progress of mild cases to recovery)	0.25
*γ*_*H1*_	Inverse of infectious period (average rate of progress of hospitalized cases to recovery)	0.1
rf	Reporting fraction	0.425

In this model, the *S* compartment represents the number of individuals susceptible to the infection, which at the start of each annual season was set to 5 million representing the total population of Ireland. The *E* compartment, representing exposed individuals, was set to zero at the start of the season, and the *I* compartment representing the infected, was set to 1 at the start of each season. The other compartments were set to zero at the start of the seasons. They represent: *H*_0_, mild cases, *H*_1_, hospitalized cases, and *R*, the recovered individuals.

New exposures occur at a rate of *λS,* where λ is the force of infection. This is calculated as $\left(I+H_{0}\right)\beta_{t}S/N$ per day, meaning that both individuals recently infectious (post latent period) and those with mild symptoms contribute to the force of infection. The parameter $\beta_{t}$ is the time-varying disease transmission rate, the estimation of which is one of the objectives of this study. Vaccination effects are incorporated into $\beta_{t}$ reflecting reduced transmission due to some immunity in the population. The average periods spent in compartments *E, H*_0,_ and *H*_1_ are given by the reciprocal of *ε* (incubation period), *γ_H0_* (recovery period), and *γ_H1_* (average length of stay), respectively. We assume that newly infectious patients exhibit mild symptoms and either stay outside the hospital (in compartment H0) or are hospitalized (in compartment H1). Hospitalization could be due to the severity of the infection or due to another cause. It is assumed that hospitalized cases are isolated and do not contribute to the force of infection. In order to transit infected individuals to the appropriate *H* compartment, the hospitalization proportion *h*, is employed along with a progression rate *δ*, the reciprocal of time from becoming infectious to hospitalization, which is set to 1. The reporting fraction, *rf*, was used to adjust the accumulated number of new hospitalized individuals entering the *H*_1_ compartment, which is equivalent to the hospitalization incidence, i.e., the seven-day smoothed cases time series, used to calibrate the parameters.

During the main part of the investigation, only the values of the piecewise-constant function $\beta_{t}$ were optimized while the remaining parameters were all kept static. Initial parameter estimates were taken from the literature (*ε*, *γ_H0_*) (33), estimated from case-based data (*h, rf*) or from hospital inpatient data (*γ_H1_*). Data from the 2023/2024 season were used to fine-tune all the parameters in the initial phase after which the determination of plausible changepoints and $\beta_{t}$ optimization was implemented. Alternative approaches for $\beta_{t}$ optimization are considered in *SI Appendix*, Text section A.

[2.1] $$\begin{array}{c} \frac{dS}{dt}=-\lambda S\\ \frac{dE}{dt}=\lambda S-\epsilon E\\ \frac{dI}{dt}=\epsilon E-\delta I\\ \frac{dH_{0}}{dt}=\delta \left(1-h\right)I-\gamma_{0}H_{0}\\ \frac{dH_{1}}{dt}=\delta hI-\gamma_{1}H_{1}\\ \frac{dR}{dt}=\gamma_{0}H_{0}+\gamma_{1}H_{1} \end{array}$$

[2.2] $$\lambda =\frac{\beta_{t}\left(I+H_{0}\right)}{N}$$

[2.3] $$N=S+E+I+H_{0}+H_{1}+R\;$$

[2.4] $$\left\{\begin{array}{ccc} \beta_{1}:t & \equiv & interval\;1\\ \beta_{2}:t & \equiv & interval\;2\\ \beta_{3}:t & \equiv & interval\;3\\ \beta_{4}:t & \equiv & interval\;4\\ \beta_{5}:t & \equiv & interval\;5 \end{array}\right.$$

### Optimization of Transmission Parameter $\beta_{t}$.

Let $\beta_{t}$ denote the time-varying transmission rate. We assume that $\beta_{t}$ is piecewise constant with an unknown number and configuration of changepoints such that for *K* transmission intervals

$$\beta_{t}\in \left\{\beta^{\left(0\right)},\cdots ,\beta^{\left(K-1\right)}\right\},\beta_{t}=\beta^{\left(j\right)}⟺t\in \left[\tau_{j},\tau_{j+1}\right),$$

where $\tau_{1},\cdots ,\tau_{K-1}$ denote changepoint locations, and the constants $\beta^{\left(j\right)}$ represent the (unknown) transmission rates associated with each interval.

In keeping with the assumed piecewise constant nature of the time-varying transmission parameter ($\beta_{t}$), the parameter was coded as a covariate in the *pomp* model in a way that up to five distinct intervals can be applied (K=5). For a given set of interval-lengths, the $\beta_{t}$ values were optimized for each interval sequentially. For example, interval one could be days 1 to 50, with interval two from day 51 to day 100 and interval three from day 101 to day 175, and in this case $\beta_{t}$ values for only three intervals would be estimated one at a time. Note that in this example, days 51 and 101 are changepoints. The process for determining the location of changepoints is crucial to this work and is discussed in the next section.

Optimization of $\beta_{t}$ for each interval was achieved through a sequential slice design approach, which takes successively narrowing cross-sections through the likelihood surface within a limited parameter space, see Algorithm 1. Following preliminary testing, an initial range of 0.01 to 4 was selected as lower and upper limits for $\beta_{t}$, and 11 evenly spaced slices were tested via trajectory matching (*SI Appendix*) against the case-based data corresponding to the interval under test. The slice value with the minimum negative log likelihood (cost, *C*) was identified; this was designated the best fit $\beta_{t}$ value in the first round. For the next round, slices immediately below and above the previous best fit were taken as revised lower and upper bounds, and 11 new slices were taken. This process was repeated until a tolerance of 1 × 10^−3^ between the limits was reached or the number of repeats exceeded a maximum of 200 iterations. The value for $\beta_{t}$ was stored along with the cost for the given interval, and the $\beta_{t}$ value for the next interval was then estimated.

Algorithm 1: Optimizing beta per time interval

**Inputs:**

- time_series_data: Time series data segmented by interval
- initial_bounds: Tuple specifying the initial search range for beta, e.g., (0.01, 4)
- num_slices: Number of beta values to evaluate per iteration (e.g., 11)
- tolerance: Minimum acceptable range width for convergence (e.g., 1e-3)
- max_iterations: Maximum number of iterations allowed (e.g., 200)

**Output:**

- best_beta: Optimal beta value per interval
- cost: Associated cost for each best_beta

**Procedure:**

For each interval in time_series_data:

i. **Initalize:**
  lower_bound ← initial_bounds[1]
  upper_bound ← initial_bounds[2]
  iteration_count ← 0
  best_beta ← NULL
ii. **Iterative Optimization:** Repeat until either:
  (upper_bound - lower_bound) < tolerance, or
  iteration_count ≥ max_iterations:
    a. Generate num_slices evenly spaced beta values between lower_bound and upper_bound.
    b. For each beta_candidate:
      Compute cost = trajectory_match(beta_candidate, current_interval_data)
    c. Identify the beta_candidate with the minimum cost → best_beta
d. **Update bounds:**
  If best_beta is not at the edge of the slice range:
  Set new bounds to the slices immediately below and above best_beta
  Else:
  Narrow the bounds conservatively around best_beta
  e. Increment iteration_count
iii. **Store Results:**
  Save best_beta and its corresponding cost for the current interval

### Iterated Filtering to Find Changepoints for Transmission Parameter $\beta_{t}$.

For each season, most likely changepoints were estimated using iterated filtering (34). Each candidate sequence of changepoints is defined as a vector of ordered integers in the range 1 to 175. Each integer corresponds to a specific day on which a changepoint may occur. A candidate sequence can include up to four changepoints, thereby dividing the timeline into a maximum of five distinct intervals. The choice of only four changepoints was derived through trial and error in the initial phase of the study, however, it was kept low to avoid overfitting.

A randomly generated set of candidate sequences was used to seed the process of identifying the most plausible changepoint configuration. From this set, the best-fitting candidates were selected based on the cost (negative log likelihood) as per Algorithm 1. For each selected candidate, a random perturbation was applied to the changepoint locations to introduce local variation and generate J new sequences, see Algorithm 2. The top K best fitting candidates from this expanded pool were again selected, and weights were calculated for each changepoint based on both its associated cost C and its frequency of occurrence across the selected candidates, as described in Eq **4.1**.

Let:

- $F^{\left(i\right)}\in {\left\{0,1\right\}}^{175}$ be the $i^{\mathrm{th}}$ binary vector,
- $C_{i}$ be the cost associated with $F^{\left(i\right)}$,
- K be the total number of vectors,
- $W_{j}$ be the final score at position j∈1,⋯,175.

Then:


[4.1] $$W_{j}=\frac{\sum_{i=1}^{K}F_{j}^{\left(i\right)}/C_{i}}{\sum_{i=1}^{K}1/C_{i}}$$

Using these weights, new candidates were generated through resampling, and a final selection was made, again using cost to find best fitting candidates. In the last step, weights were recalculated to emphasize the most likely changepoint locations. These steps were iterated several times for each season resulting in a vector of mean weights, which represents the overall likelihood of a particular day in the time series being a plausible changepoint.

The following meta-parameters were applied: initially 100 random candidates with the top 20 best fitting being selected for the perturbation step. In this step, each selected candidate was expanded 10 times, by perturbing any changepoint by up to four days either side of the original or deleted with a probability of 0.2. Along with the original, unmutated candidates, this yielded a pool of up to J=220 unique candidates, the top 20 best fitting of which were again selected. Duplicate candidates were eliminated at each stage. After the first weight calculation step, 100 new candidates were generated through weighted sampling, and again, the top 20 were selected. From this, the second weight calculation yielded a vector for a single iteration. The mean weights from 50 iterations per each season were taken for the final analysis.

Algorithm 2: Iterated filtering for changepoint detection

**Inputs:**

- time_series_data: Observed data over time
- max_changepoints: Maximum number of changepoints allowed (e.g., 4)
- total_days: Total number of days in the time series (e.g., 175)
- num_initial_candidates: Number of initial changepoint sequences (e.g., 100)
- num_selected: Number of top-performing candidates to retain (e.g., 20)
- perturbation_expansions: Number of perturbations per selected candidate (e.g., 10)
- perturbation_range: Range of days for perturbation (e.g., ±4 d)
- deletion_prob: Probability of deleting a changepoint during perturbation (e.g., 0.2)
- num_resampled_candidates: Number of candidates via resampling (e.g., 100)
- num_iterations: Total number of filtering iterations (e.g., 50)

**Output:**

mean_weight_vector: A vector of length total_days representing the likelihood of each day being a changepoint

i. **Initialization:**
  Set mean_weight_vector ← zero vector of length total_days
ii. **Iterative Filtering (Repeat for num_iterations):**
  **Step 1: Generate Initial Candidates**
  candidates ← generate_random_candidates(num_initial_candidates, max_changepoints, total_days)
  **Step 2: Evaluate and Select Top Candidates**
  costs ← evaluate_candidates(candidates, time_series_data)
  selected_candidates ← select_top_candidates(candidates, costs, num_selected)
  **Step 3: Local Exploration via Perturbation**
  For each candidate in selected_candidates:
  Generate perturbation_expansions perturbed versions using:
  Random shifts within perturbation_range
  Random deletions with probability deletion_prob
  perturbated_candidates ← all generated perturbations
  all_candidates ← remove_duplicates(selected_candidates + perturbated_candidates)
  **Step 4: Reevaluate and Select Top Candidates**
  costs ← evaluate_candidates(all_candidates, time_series_data)
  selected_candidates ← select_top_candidates(all_candidates, costs, num_selected)
  **Step 5: Calculate Changepoint Weights as per** (Eq **4.1**)
  weights ← calculate_weights(selected_candidates, costs, total_days)
  **Step 6: Resample Candidates Based on Weights**
  resampled_candidates ← resample_candidates(weights, num_resampled_candidates, max_changepoints)
  **Step 7: Evaluate Resampled Candidates**
  costs ← evaluate_candidates(resampled_candidates, time_series_data)
  selected_candidates ← select_top_candidates(resampled_candidates, costs, num_selected)
  **Step 8: Final Weight Update**
  iteration_weights ← calculate_weights(selected_candidates, costs, total_days)
  mean_weight_vector ← mean_weight_vector + iteration_weights
iii. **Postprocessing:**
  mean_weight_vector ← mean_weight_vector/num_iterations
  **Return:**
  mean_weight_vector

### Cross-Seasonal Changepoint Analysis.

To explore the consistency and variability of inferred changepoint locations across different seasons, we conducted a cross-seasonal analysis for the four seasons. We first constructed kernel density estimates (KDE, Gaussian kernel with bandwidth of 4) of the normalized mean weights resulting from the 50 iterations in Algorithm 2 for each season. Local maxima of the observed KDE were compared across the seasons. For each season the four highest-density local maxima were used to generate 150 new sequences by perturbation around the maxima (perturbation process described above, with a deletion probability of 0.6) and the best fitting trajectory for each season was used to visualize model fit against the observed time series data, and to extract corresponding changepoints and $\beta_{t}$ values. These outputs are presented across all seasons studied to highlight shared and divergent temporal structures. The top 20 best fitting trajectories’ cost values for season were also used to compare cost values from different stages of the analysis.

Finally, a universal KDE obtained by combining the normalized mean weights from all the seasons was calculated. The four highest-density local maxima were assumed to be universal changepoints for all seasons. Trajectories for each season were calculated optimizing the $\beta_{t}$ values against the time series for each season individually.

## Supplementary Material

Appendix 01 (PDF)

## Data Availability

Anonymized csv data, dates, and counts data have been deposited in Github (https://github.com/AjayOza/epi_changepoints) (35).

## References

[1] D. Balcan , Seasonal transmission potential and activity peaks of the new influenza A (H1N1): A Monte Carlo likelihood analysis based on human mobility. BMC Med. **7**, 45 (2009). 19744314 10.1186/1741-7015-7-45 PMC2755471

[2] ECDC, Surveillance for seasonal influenza in Europe–ECDC Technical Report. (2017). https://www.ECDCecdc.europa.eu/en/publications-data/surveillance-seasonal-influenza-europe.

[3] HSE-HPSC, Influenza surveillance in Ireland: Annual report. (2024). https://www.hpsc.ie/a-z/respiratory/influenza/surveillance/. [cited 2025 15/102/025].

[4] G. M. Abernethy, D. H. Glass, Optimal COVID-19 lockdown strategies in an age-structured SEIR model of Northern Ireland. J. R. Soc. Interface **19**, 20210896 (2022). 35259954 10.1098/rsif.2021.0896 PMC8905176

[5] J. M. Mendes, P. S. Coelho, Addressing hospitalisations with non-error-free data by generalised SEIR modelling of COVID-19 pandemic. Sci. Rep. **11**, 19617 (2021). 34608201 10.1038/s41598-021-98975-w PMC8490474

[6] J. Dureau, K. Kalogeropoulos, M. Baguelin, Capturing the time-varying drivers of an epidemic using stochastic dynamical systems. Biostatistics **14**, 541–555 (2013). 23292757 10.1093/biostatistics/kxs052

[7] J. A. Bouman , Bayesian workflow for time-varying transmission in stratified compartmental infectious disease transmission models. PLoS Comput. Biol. **20**, e1011575 (2024). 38683878 10.1371/journal.pcbi.1011575 PMC11081492

[8] A. Spannaus , Inferring the spread of COVID-19: The role of time-varying reporting rate in epidemiological modelling. Sci. Rep. **12**, 10761 (2022). 35750796 10.1038/s41598-022-14979-0 PMC9232503

[9] J. P. Gleeson , Calibrating COVID-19 susceptible-exposed-infected-removed models with time-varying effectivecontact rates. Philos. Trans. A Math. Phys. Eng. Sci. **380**, 20210120 (2022). 34802273 10.1098/rsta.2021.0120 PMC8607149

[10] X. Pang , Time-varying reproduction number estimation: Fusing compartmental models with generalized additive models. J. R. Soc. Interface **22**, 20240518 (2025). 39878127 10.1098/rsif.2024.0518 PMC11776018

[11] Y. Liu , Evaluating effects of dynamic interventions to control COVID-19 pandemic: A case study of Guangdong, China. Int. J. Environ. Res. Public Health. **19**, 10154 (2022). 36011787 10.3390/ijerph191610154 PMC9407938

[12] UKHSA, GP out-of-hours syndromic surveillance system bulletin (England). (2025). https://assets.publishing.service.gov.uk/media/695512a597f1c5e3c92a8365/UKHSAUKHSA-GP-out-of-hours-syndromic-surveillance-bulletin-2025-week-52.pdf.

[13] WHO, Global respiratory virus activity weekly update: Update No. 559. (2025). https://cdn.who.int/media/docs/default-source/influenza/influenza-updates/2025/202552_who-respiratory-virus-update_559.pdf?sfvrsn=7c652705_3&download=true. [cited 2026 03/03/2026].

[14] Y. Singh, Robust scaling: Why and how to use it to handle outliers. Proclus Academy (2022). https://proclusacademy.com/blog/robust-scaler-outliers/. Accessed 1 May 2026.

[15] X. Liu , Improving greedy local search methods by switching the search space. Appl. Intell. **53**, 22143–22160 (2023).

[16] B. Chen , Prediction of an epidemic spread based on the adaptive genetic algorithm. Front. Phys. **11**, 1195087 (2023).

[17] P. Coletti , Shifting patterns of seasonal influenza epidemics. Sci. Rep. **8**, 12786 (2018). 30143689 10.1038/s41598-018-30949-x PMC6109160

[18] S. L. Jegede, K. J. Szajowski, Change-point detection in homogeneous segments of COVID-19 daily infection. Axioms **11**, 213 (2022).

[19] W. Wickramaarachchi, S. Perera, An SIER model to estimate optimal transmission rate and initial parameters of COVD-19 dynamic in Sri Lanka. Alex. Eng. J. **60**, 1557–1563 (2021).

[20] P. V. Savi, M. A. Savi, B. Borges, A mathematical description of the dynamics of Coronavirus Disease 2019 (COVID-19): A case study of Brazil. Computat. Math. Methods Med. **2020**, 9017157 (2020). 10.1155/2020/9017157 PMC7528041 33029196

[21] National Near-Patient Testing (NPT) Consultative Group, Guidelines for safe and effective near-patient testing (NPT). Royal College of Physicians of Ireland, Faculty of Pathology, Dublin, Ireland (2022). https://www.rcpi.ie/Portals/0/Document%20Repository/Faculty%20of%20Pathology/FPath_About_Guidelines%20for%20safe%20and%20effective%20near-patient%20testing_2022.pdf. Accessed 1 May 2026.

[22] A. McKenna , Respiratory virus testing practices in acute hospital settings; results from a national laboratory survey in Ireland, July/August 2023. Epi-Insight (2024). https://ndsc.newsweaver.ie/4otaa688p3/17t8xqihvu0.

[23] L. Marron , An evaluation of the severe acute respiratory infection surveillance system in Ireland. BMC Public Health **25**, 492 (2025). 39915743 10.1186/s12889-025-21645-3 PMC11800475

[24] ECDC, Sentinel surveillance. (2023). https://www.ecdc.europa.eu/en/seasonal-influenza/surveillance-and-disease-data/facts-sentinel-surveillance. [cited 2025 16/10/2025].

[25] HSE-HPSC, Sentinel GP surveillance of clinical diseases. (2023). https://www.ecdc.europa.eu/en/seasonal-influenza/surveillance-and-disease-data/facts-sentinel-surveillance. [cited 2025 16/10/2025].

[26] J. Shaman, A. Karspeck, Forecasting seasonal outbreaks of influenza. Proc. Natl. Acad. Sci. U.S.A. **109**, 20425–20430 (2012). 23184969 10.1073/pnas.1208772109 PMC3528592

[27] A. Gonzales, G. Guruswamy, S. R. Smith, Synthetic data in health care: A narrative review. PLOS Digit. Health **2**, e0000082 (2023). 36812604 10.1371/journal.pdig.0000082 PMC9931305

[28] A. L. Bertozzi , The challenges of modeling and forecasting the spread of COVID-19. Proc. Natl. Acad. Sci. U.S.A. **117**, 16732–16738 (2020). 32616574 10.1073/pnas.2006520117 PMC7382213

[29] M.-A. Rizoiu, S. Mishra, Q. Kong, M. Carman, L. Xie, “SIR-Hawkes: Linking epidemic models and Hawkes processes to model diffusions in finite populations” in *Proceedings of the 2018 World Wide Web Conference*, P.-A. Champin, F. Gandon, M. Lalmas, P. G. Ipeirotis, Eds. (ACM, New York, NY, 2018), pp. 419–428.

[30] HSE-HPSC, Respiratory virus notification data hub.HSE-HPSC. Respiratory Virus Notification Data Hub. (2025). https://respiratoryvirus.hpsc.ie/. [cited 2025 16/10/2025].

[31] Health Atlas Ireland, Health Atlas Ireland. (2025). https://www.healthatlasireland.ie/. [cited 2025 16/10/2025].

[32] A. A. King, D. Nguyen, E. L. Ionides, Statistical inference for partially observed markov processes via the R package pomp. J. Stat. Softw. **69**, 1–43 (2016).

[33] F. Brauer, C. Castillo-Chavez, Z. Feng, “Models for Influenza” in Mathematical Models in Epidemiology, F. Brauer, C. Castillo-Chavez, Z. Feng, Eds., (Springer, New York, NY, 2019), pp. 311–350.

[34] E. L. Ionides, C. Bretó, A. A. King, Inference for nonlinear dynamical systems. Proc. Natl. Acad. Sci. U.S.A. **103**, 18438–18443 (2006). 17121996 10.1073/pnas.0603181103 PMC3020138

[35] A. N. Oza, epi_changepoints: Change point detection for epidemiological time series. GitHub. https://github.com/AjayOza/epi_changepoints. Deposited 23 April 2026.

