# Supplementary material for: Detecting changepoints in dynamical systems: Modeling time-varying transmission of seasonal influenza
Source: Proc Natl Acad Sci U S A. 2026 May 19;123(21):e2533861123. doi: 10.1073/pnas.2533861123 (PMC13213938; doi:10.1073/pnas.2533861123)
Supplement: Supplementary file 1 — Appendix 01 (PDF) [file pnas.2533861123.sapp.pdf]

Supplementary Information Text

PAPER: Detecting changepoints in dynamical systems: Modelling time-varying transmission of seasonal influenza, by Oza *et al.*

Section A: Optimisation Methods

In this paper, we apply the methods developed by King *et al.* as described in their educational material for the R pomp package (Simulation-based Inference for Epidemiological Dynamics). The compartmental model utilised is a Markov model that updates the state vector at each time step based solely on the current state and transition parameters, which includes the transmission rate β_t. Consequently, the initial state of interval $k+1$ depends upon the final state of interval k; adjusting β during interval k modifies the compartment counts entering interval $k+1$. As such, it is not feasible to optimise β_{k+1} prior to determining the accurate state generated by β_k.

Simultaneous multi-dimensional optimisation techniques, such as particle swarm optimisation, tend to be unstable and inefficient computationally in this application. Moreover, sequential estimation aligns with the inherent temporal structure of epidemic progression. Consequently, our approach involves optimising β_t across multiple piecewise-constant intervals in a sequential fashion.

A slice-design mechanism is specifically employed to optimise each interval sequentially. Alternative approaches, including Nelder-Mead and Brent, are accessible through the *optim* function in R for one-dimensional optimisation. However, relying exclusively on these *optim* methods may pose limitations in stochastic models due to these models' propensity for ill-conditioning.

Section B: Interpreting Changepoints in Time-Varying Transmission Models

1. Introduction

In this paper the transmission rate β_t is estimated as a time-varying parameter to capture changes in disease dynamics. The approach is to represent β_t as a piecewise-constant function, allowing it to change at a set of inferred changepoints. This method is flexible and computationally tractable, but it raises an important interpretive challenge: not every detected changepoint corresponds to a real intervention or meaningful event.

Some changes in β_t may reflect genuine abrupt changes such as planned interventions or policy measures, while others may arise from more gradual seasonal or behavioural trends that the piecewise-constant model approximates with instantaneous "steps". Additional changepoints may simply reflect statistical noise or limitations in the modelling approach.

Understanding which changepoints matter is crucial. Misinterpreting natural variability as an intervention effect, or overlooking genuine structural changes, can lead to incorrect conclusions about the drivers of epidemic trends.

This section outlines the nature of the problem, provides a demonstration using synthetic data, and then discusses some practical options for interpreting changepoints responsibly.

2. The Core Challenge

A piecewise-constant β_t approximation divides the epidemic trajectory into discrete intervals. However, the epidemic process itself can be smooth and continuous, influenced by factors such as behaviour, environment, or seasonality. When a smooth trend is approximated by a step function, the model will introduce artificial changepoints. As a result:

- The algorithm may introduce "changepoints" simply to follow a smooth curve.
- These algorithmic changepoints do not automatically correspond to meaningful epidemiological events.
- Conversely, a real intervention may or may not coincide with a fitted changepoint, depending on the method, the noise level, and model flexibility.

This issue arises whether the true β_t is smoothly varying or discontinuous and is especially important when interpreting changepoints as indicators of policy impact or behavioural shifts. The synthetic examples used in this section using regular sinusoidal curves are unlikely to occur in the real-life situations that provide the context of this paper. More likely, irregular changes in β_t may drive the model used to fit real data. However, it is possible that during certain time periods the β_t function may vary sinusoidally to some extent with abrupt changes resulting from interventions or other meaningful events. Certainly, there are examples of sigmoidal fits to interventions as mentioned in the main article (Liu, Y., *et al*, 2022).

3. Worked Example Using Synthetic Data

To better understand the limitations of our method, we generated synthetic infection trajectories from β_t values of both uninterrupted and intervened sinusoidal curves. The

changepoint algorithm mentioned in the main article was used to generate the piecewise-constant β_t.

1. An uninterrupted sinusoidal curve for β_t was generated using equation S1 and another curve with an intervention was created with equation S2.
2. Trajectories were generated using the same parameters used for the *pomp* compartmental model as the ones used in the main article.
3. The synthetic trajectories (corresponding to reported cases in the main article) were modelled using the changepoint algorithm.

The optimal piecewise-constant β_t values are shown alongside the trajectories of one uninterrupted example (Figure S1) and one example with an intervention (Figure S2). The following equations were used to create the examples.

$y(x) = A + B \sin\left(\frac{2\pi(x-\phi)}{T}\right)$	(Eq S1)
$y(x) = \begin{cases} c, & t_0 \le x < t_1, \\ A + B \sin\left(\frac{2\pi(x-\phi)}{T}\right), & \text{otherwise} \end{cases}$	(Eq S2)

The parameters and values used were (for both equations):

A mean level of the sinusoid (0.26),

B amplitude (0.20),

T period (175),

ϕ phase shift (0),

x days (1 to 175)

For equation S2 only:

c intervention value (0.25),

t_0 and t_1 define the intervention window (75 to 100)

This analysis illustrates that it is possible for the changepoint algorithm to produce a series of (piecewise-constant) β_t values such that a realistic trajectory can be fitted to synthetic data that were generated using sinusoidal β_t values. Changepoints were introduced by the algorithm to follow the curve as expected. However, note in Fig. S1b that areas of the β_t values of the sinusoidal curve that do not produce large changes in the reported cases (at the beginning and the end of the epidemic) produced fewer changepoints. Areas corresponding to the more dynamic or acute phase of the epidemic produced more changepoints. In this way, the changepoint algorithm did not generate the β_t values that fit a sinusoidal curve in a fashion a smoothing algorithm would. These changepoints can be understood as “natural”.

The sinusoidal curve of β_t values that introduced an intervention produced a time series trajectory very different from the uninterrupted one (Fig. S2). A changepoint corresponding to the intervention point (day 75) was produced by the algorithm. The β_t values of 0.241 during

the period of intervention (days 75 to 100) closely matched the introduced value of 0.25 in this example. This changepoint can be understood as “interventional”.

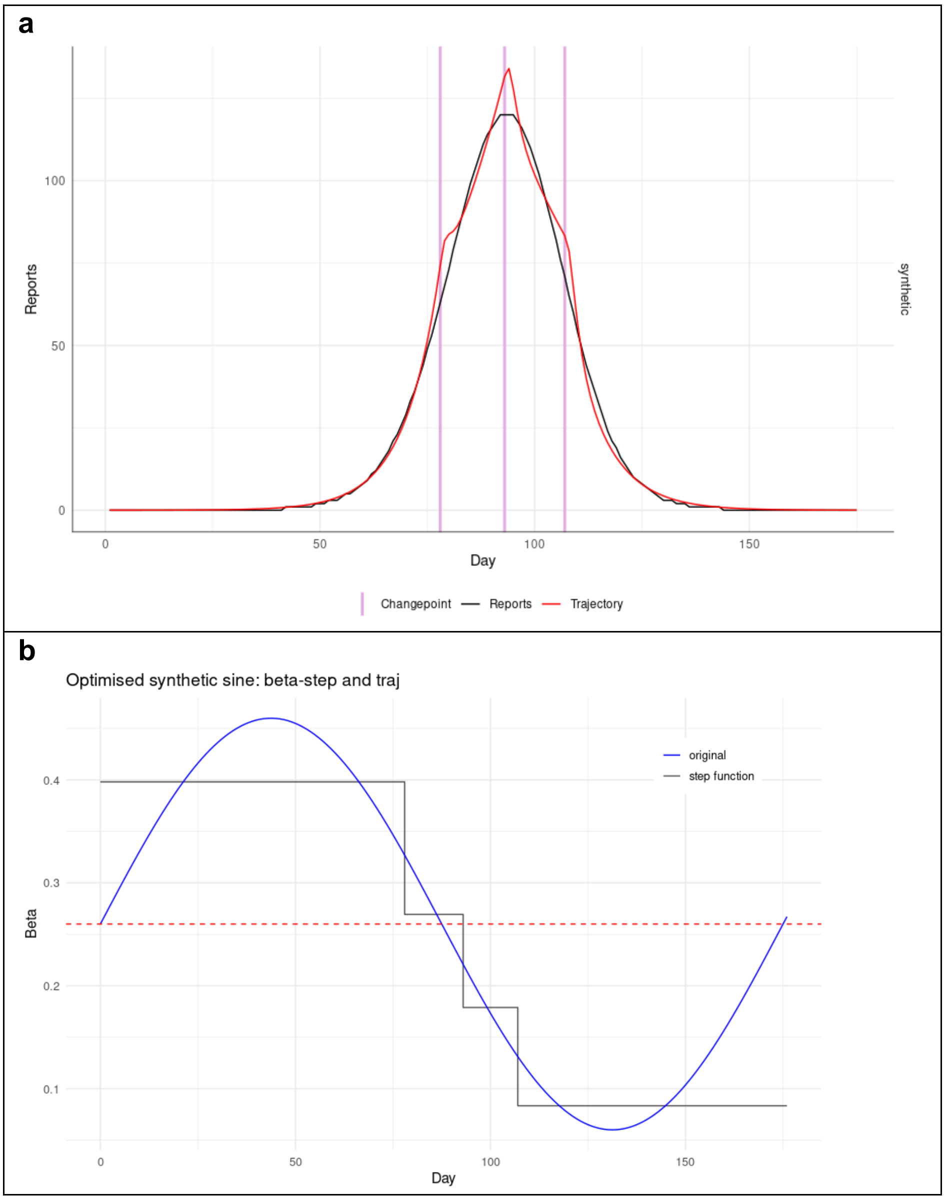

Figure S1. Uninterrupted example. **a.** Time series of reports and best fitting modelled trajectory of infections from the changepoint determination algorithm showing relative positions of changepoints. **b.** The piecewise-constant inferred β_t (grey) of the fitted trajectory along with the original sinusoidal function (blue).

Now note that although all changepoints thus derived are relevant, the “interventional” changepoint cannot be readily distinguished from the “natural” changepoints from the step function alone. This illustrates the core issue.

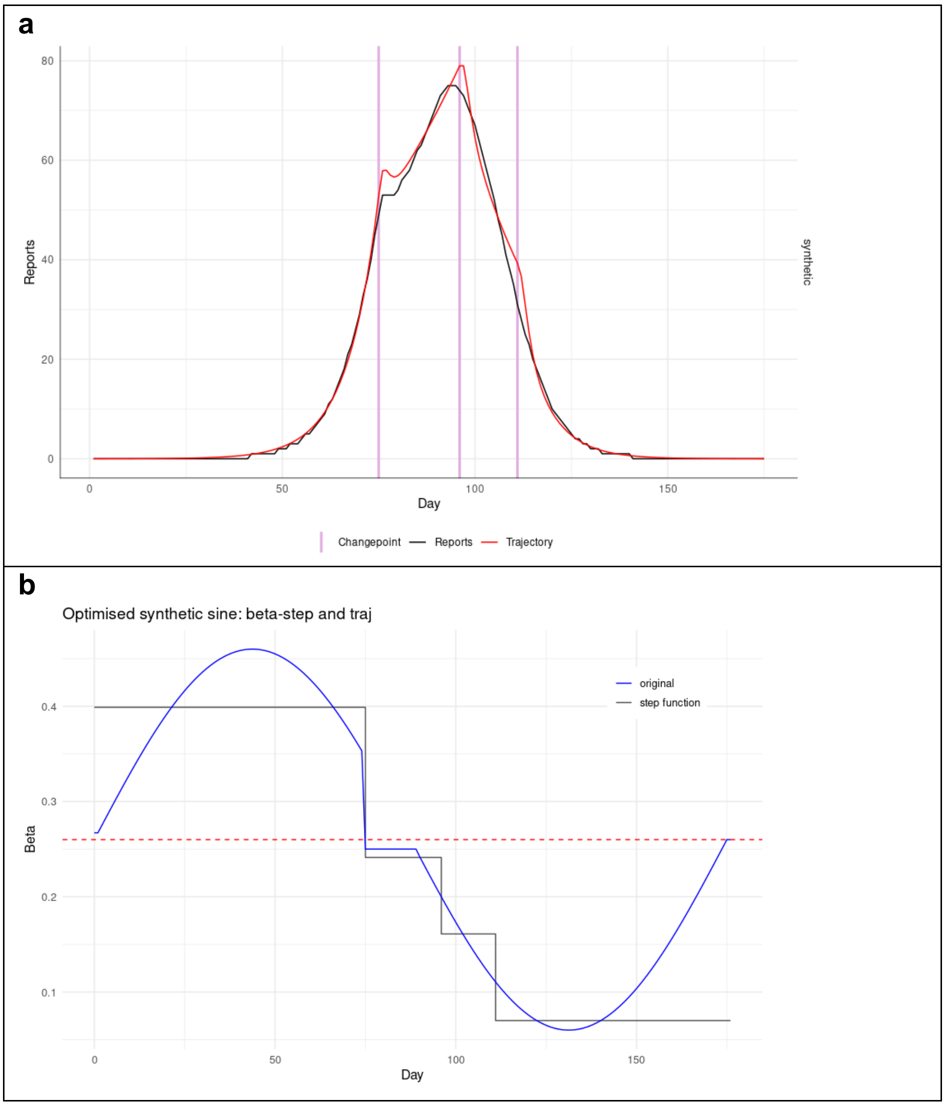

Figure S2. Example with intervention between days 75 and 100, when the beta was set to 0.25. **a.** Time series of reports and best fitting modelled trajectory of infections from the changepoint determination algorithm showing relative positions of changepoints. **b.** The piecewise-constant inferred β_t (grey) of the fitted trajectory along with the original sinusoidal function (blue).

4. Options for Interpreting Changepoints

The results above demonstrate the need for a structured approach to interpretation.

4.1. Treat Raw Changepoints as Descriptive Only

The piecewise-constant β_t changepoints should be viewed initially as descriptive features of the fitted approximation, not as indicators of underlying causal events. Their primary role is to show where the step estimator needed additional flexibility to capture curvature or changes in the signal. Consequently, the presence of a changepoint alone does not indicate an intervention effect.

4.2. When a Specific Intervention Date is Known, Conduct a Targeted Test

If an intervention occurs at a known time, that date should be evaluated explicitly using interrupted time-series (ITS) or similar methods. The usage of the changepoint detection

algorithm alone to reveal the intervention should be avoided; instead this method should be used to support ITS or similar approaches. Validation checks (such as placebo dates or model comparison) can be employed to confirm whether the observed deviation is more consistent with an intervention effect than it is with natural variation.

4.3. When No Intervention Dates Are Known, Use Additional Tests

If the objective is exploratory (for example, identifying unknown structural changes in transmission, search for shifts in level or trend that cannot be explained by the smooth background alone), then the piecewise-constant changepoint detection algorithm provides potential points to test.

However, as before, changepoints identified at this stage should be considered candidate structural changes, not confirmed interventions. If a changepoint aligns with contextual information (such as changes in behaviour, reporting practices, or unrecorded interventions), a follow-up ITS-style analysis can be used to evaluate its credibility. Again, methods described in this paper can easily be employed to construct a counterfactual.

This two-step process helps avoid confusing natural seasonal patterns with abrupt epidemiological events.

5. Summary

Piecewise-constant β_t models are convenient, but their changepoints do not automatically correspond to real events. Without appropriate analysis, it is difficult to determine whether a shift reflects an intervention, natural variation, or algorithmic approximation.

Options include the use of interrupted time-series or similar as supporting tests; treating changepoints as potential (rather than confirmatory) candidate that may merit further investigation, especially if aligning with contextual information; and comparing observed changes to a modelled counterfactual.

The options provided are neither prescriptive nor exhaustive. Interpretation of the changepoints determined in the main article was beyond the scope of the study, therefore this text only offers a general discussion of the conceptual steps required to implement confirmatory testing and highlights the importance of using principled methods for interpreting changepoints. The main article leaves the interpretation as descriptive only.
